# Life-Threatening Hypertriglyceridemia in a Patient on Ruxolitinib and Sirolimus for Chronic Graft-versus-Host Disease

**DOI:** 10.1155/2018/4539757

**Published:** 2018-11-04

**Authors:** Allison P. Watson, Claudio G. Brunstein, Shernan G. Holtan

**Affiliations:** Blood and Marrow Transplant Program, Department of Medicine, University of Minnesota, Minneapolis, MN, USA

## Abstract

Ruxolitinib is an oral selective Janus-associated kinase 1 (JAK1) and JAK2 inhibitor that was initially approved by the FDA in 2014 for treatment of myelofibrosis. In preclinical and retrospective clinical studies, use of ruxolitinib was shown to reduce graft-versus-host-disease (GVHD) in allograft recipients with moderate/severe corticosteroid-dependent or refractory chronic GVHD. While the exact mechanism for action in GVHD is not yet fully understood, prospective studies are ongoing and some patients are receiving ruxolitinib in the setting of steroid refractory GVHD. Although ruxolitinib is generally well tolerated, here we describe a case involving a 50-year-old man with acute myeloid leukemia and chronic GVHD who experienced life-threatening hypertriglyceridemia associated with concomitant use of sirolimus and ruxolitinib for GVHD. This case report highlights the importance of vigilance for severe side effects in novel immunosuppressive drug combinations.

## 1. Introduction

Ruxolitinib is an oral selective Janus-associated kinase 1 (JAK1) and JAK2 inhibitor that was initially approved by the FDA in 2014 for treatment of myelofibrosis. In preclinical and retrospective clinical studies, use of ruxolitinib was shown to reduce graft-versus-host-disease (GVHD) in allograft recipients with moderate/severe corticosteroid-dependent or refractory chronic GVHD [1–3]. Activated JAKs are required for T-effector cell responses in inflammation, and their blockade is thought to suppress the proinflammatory signaling that mediates tissue damage in GVHD [4]. While prospective studies are ongoing, some patients are receiving ruxolitinib in the setting of steroid refractory GVHD [5]. Although ruxolitinib is generally well tolerated [6], here we describe a case of life-threatening hypertriglyceridemia associated with concomitant use of sirolimus and ruxolitinib for GVHD.

## 2. Case Report

The patient is a 50-year-old man with history of myelodysplastic syndrome, which had progressed to acute myeloid leukemia. He underwent nonmyeloablative allogeneic hematopoietic stem cell transplant (HSCT) in 2013 and had numerous complications. He had graft failure and underwent a successful second allogeneic sibling peripheral blood stem cell transplant in 2014. His disease course was complicated by chronic GVHD (cGVHD) involving the eyes, skin, liver, and buccal mucosa.

For his cGVHD, he had been treated with prednisone on a taper, sirolimus, and twice weekly extracorporeal photopheresis (ECP). However, he developed a systemic infection with* Mycobacterium abscessus* (*M. abscessus*) and the ECP Vortex Port® had to be removed. As* M. abscessus* requires prolonged antibiotic therapy, we elected not to replace the port and he was switched to ruxolitinib, used in combination with sirolimus and prednisone. He was seen in clinic for evaluation of severe fatigue, aching abdominal pain localized to the upper abdomen radiating into the back, headache, and nausea. He noted diaphoresis, but no fevers. Initial lab work-up was notable only for albumin 2.4, alkaline phosphatase 100, ALT 105, AST 105, WBC 4.6, hemoglobin 14.2, and platelets 202. Blood sample was lipemic and triglycerides were >4000 (ref. range <150 mg/dL). Subsequent labs included amylase 12 (ref. range 30-110 U/L) and lipase 225 (ref. range 73-393 U/L). CT of the abdomen and pelvis with intravenous contrast showed no convincing evidence of pancreatitis, only a small calcific focus at the tail of the pancreas, representing either a parenchymal calcification or a small ductal stone. He was admitted to the hospital for further work-up and management.

On admission, medications included acyclovir, amlodipine, ascorbic acid, calcium carbonate, cefoxitin, docusate, fluconazole, furosemide, gabapentin, lisinopril, methadone, metoprolol tartrate, multivitamin, omega 3 fatty acids, pantoprazole, pravastatin, prednisone, ruxolitinib, sirolimus, sulfamethoxazole-trimethoprim, tamsulosin, tigecycline, trazodone, venlafaxine, cholecalciferol, and zinc acetate.

**Figure 1:**
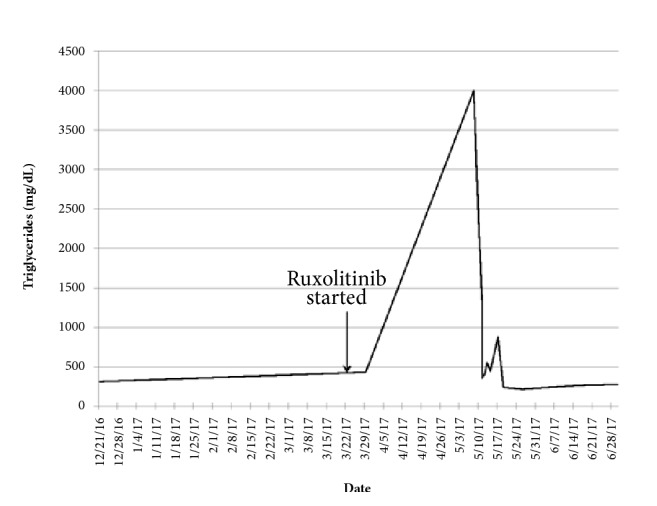
Serum triglyceride level with relation to starting ruxolitinib for chronic GVH.

While hospitalized, sirolimus and lisinopril were held and endocrinology was consulted for evaluation of hypertriglyceridemia. Prior triglyceride levels were noted to be between 300 and 400 over the last several years. He was started on an insulin drip with a goal to reduce triglyceride level to less than 500 mg/dL. The patient's abdominal pain improved slightly. Triglyceride levels trended down from 2983 (on hospital arrival) to 2228 (on hospital day 1 after insulin) and 2093 (on hospital day 2) (see Figure 1). Therapeutic plasma exchange (TPE) was then planned to bring down the triglyceride level quickly and prevent any further sequelae of marked hypertriglyceridemia. Ruxolitinib was also stopped at that time. TPE was performed on hospital day 3 and subsequently triglyceride level decreased to 1305 and 407 on hospital day 4. He noted resolution of his abdominal discomfort with improved triglyceride levels.

The patient was instructed to follow a low-fat and low-carbohydrate diet after hospital discharge. Triglyceride levels remained high (446-881 mg/dL) for the week following discharge, but trended down to the 200s within two weeks of discharge (211-277 mg/dL) and remained within normal or minimally elevated ranges (94-270 mg/dL) for the following year. After stopping ruxolitinib, the patient's GVHD was managed with prednisone, ibrutinib, topical tacrolimus, dexamethasone oral solution, pancreatic enzymes, and artificial tears.

## 3. Discussion

We hypothesize that the marked hypertriglyceridemia was secondary to the combination of sirolimus and ruxolitinib. Dyslipidemia related to sirolimus has been well-documented in the literature [7], and we had previously noted hypertriglyceridemia while the patient was on sirolimus alone in the past (205-311 mg/dL), with previously normal triglyceride levels (118-134 mg/dL) prior to sirolimus. A post hoc analysis of the data from the COMFORT-I clinical trial assessed the effects of ruxolitinib treatment on measures of metabolic and nutritional status. The overwhelming majority (96.8%) of patients in the ruxolitinib treatment group experienced an increase in total cholesterol, with a percentage increase from baseline of 26.4% versus a mean decrease of 3.3% in the placebo group [8]. It was hypothesized that this was related to a clinically meaningful gain in lean body mass in patients treated with ruxolitinib. There is very little clinical data available regarding the effect of ruxolitinib on triglyceride levels, though the package insert for Jakafi® (trade name) cites data from the RESPONSE trial in which low-grade elevations in cholesterol, triglyceride, ALT, and AST levels were observed with ruxolitinib but were not associated with clinical outcomes [9]. It is recommended to assess lipid parameters at 8-12 weeks after initiating ruxolitinib and treat according to clinical guidelines for the management of hyperlipidemia [10].

## 4. Conclusions

For many patients with hematologic diseases, allogeneic HSCT offers a potentially curative treatment option. However, many patients develop serious complications such as GVHD, which is a leading cause of nonrelapse mortality [11]. While corticosteroids remain first-line treatment for acute and chronic GVHD, some patients will become corticosteroid-dependent or have corticosteroid-refractory GVHD [12, 13]. There are currently no FDA-approved medications for corticosteroid-refractory acute GVHD, though there are ongoing prospective clinical trials which are still actively recruiting patients to evaluate the JAK1/JAK2 inhibitor ruxolitinib in patients with corticosteroid-refractory acute (REACH 1 and REACH 2) or chronic GVHD (REACH 3) [14]. Our report highlights the importance of vigilance for severe side effects in novel immunosuppressive drug combinations.
